# Irinotecan plus S-1 versus S-1 in patients with previously treated recurrent or metastatic esophageal cancer (ESWN 01): a prospective randomized, multicenter, open-labeled phase 3 trial

**DOI:** 10.1186/s40880-019-0359-7

**Published:** 2019-04-02

**Authors:** Jing Huang, Binghe Xu, Ying Liu, Junxing Huang, Ping Lu, Yi Ba, Lin Wu, Yuxian Bai, Shu Zhang, Jifeng Feng, Ying Cheng, Jie Li, Lu Wen, Xianglin Yuan, Changwu Ma, Chunhong Hu, Qingxia Fan, Xi Wang

**Affiliations:** 1 Department of Medical Oncology, National Cancer Center/National Clinical Research Center for Cancer/Cancer Hospital, Chinese Academy of Medical Sciences and Peking Union Medical College, No 17 Panjiayuan Nanli, Chaoyang District, Beijing, 100021 P. R. China; 2 Department of Medical Oncology, Henan Cancer Hospital, Zhengzhou, 450008 Henan P. R. China; 3 Department of Medical Oncology, Taizhou People’s Hospital, Taizhou, 225300 Jiangsu P. R. China; 4 Department of Medical Oncology, First Affiliated Hospital of Xinxiang Medical University, Xinxiang, 453100 Henan P. R. China; 5 Department of Medical Oncology, Tianjin Cancer Hospital, Tianjin, 300060 P. R. China; 6 Department of Medical Oncology, Hunan Cancer Hospital, Changsha, 410006 Hunan P. R. China; 7 Department of Medical Oncology, Harbin Medical University Cancer Hospital, Harbin, 150040 Heilongjiang P. R. China; 8 Department of Medical Oncology, Shandong Cancer Hospital, Jinan, 250117 Shandong P. R. China; 9 Department of Medical Oncology, Jiangsu Cancer Hospital, Nanjing, 210009 Jiangsu P. R. China; 10 Department of Medical Oncology, Jilin Cancer Hospital, Changchun, 130012 Jilin P. R. China; 11 Department of Radiotherapy, Shanxi Cancer Hospital, Taiyuan, 030013 Shanxi P. R. China; 12 Department of Medical Oncology, Shanxi Cancer Hospital, Taiyuan, 030013 Shanxi P. R. China; 13 Department of Medical Oncology, Tongji Hospital, Wuhan, 430030 Hubei P. R. China; 14 Department of Medical Oncology, Chifeng Municipal Hospital, Chifeng, 024000 Inner Mongolia P. R. China; 15 Department of Oncology, The Second Xiangya Hospital of Central South University, Changsha, 410011 Hunan P. R. China; 16 Department of Oncology, The First Affiliated Hospital of Zhengzhou University, Zhengzhou, 450052 Henan P. R. China

**Keywords:** Esophageal squamous cell carcinoma, Recurrent, Metastasis, Multicenter, open-label, randomized trial, Irinotecan, S-1, Overall survival, Progression-free survival, Objective response rate, Disease control rate

## Abstract

**Background:**

The benefit of systemic treatments in esophageal squamous cell carcinoma (ESCC) which has progressed after chemotherapy is still uncertain and optimal regimens based on randomized trials have not yet been established. We aimed to compare the efficacy of irinotecan plus S-1 with S-1 monotherapy in recurrent or metastatic ESCC patients who had resistance to platinum- or taxane-based chemotherapy.

**Methods:**

We conducted a prospective randomized, multicenter, open-label, phase 3 trial in 15 centers across China. Eligible patients were adults with histologically confirmed recurrent or metastatic ESCC, and were randomly assigned (ratio, 1:1) to receive either irinotecan plus S-1 (intravenous infusion of irinotecan [160 mg/m^2^] on day 1 and oral S-1 [80–120 mg] on days 1–10, repeated every 14 days) or oral S-1 monotherapy (80–120 mg/day on days 1–14, repeated every 21 days) using a central computerized minimization procedure. The primary endpoint was progression-free survival (PFS).

**Results:**

Between December 23, 2014 and July 25, 2016, we screened 148 patients and randomly assigned 123 patients to receive either irinotecan plus S-1 regimen (*n *= 61) or S-1 monotherapy (*n* = 62). After a median follow-up of 29.2 months (95% confidence interval [CI] 17.5–40.9 months), the median PFS was significantly longer in the irinotecan plus S-1 group than in the S-1 monotherapy group (3.8 months [95% CI 2.9–4.3 months] vs. 1.7 months [95% CI 1.4–2.7 months], hazard ratio = 0.58, 95% CI 0.38–0.86, *P* = 0.006). The objective response rates were 24.6% in the irinotecan plus S-1 group and 9.7% in the S-1 monotherapy group (*P* = 0.002). The patients in the irinotecan plus S-1 group presented with increased rates of grade 3–4 leukopenia (16.4% vs. 0%), neutropenia (14.8% vs. 1.6%), and nausea (4.9% vs. 0%). No significant difference in grade 3–4 diarrhea and no treatment-related deaths were observed in both groups.

**Conclusions:**

The combination of irinotecan with S-1 was similarly tolerable but significantly prolonged PFS compared to S-1 monotherapy as a second- or third-line treatment in patients with recurrent or metastatic ESCC.

*Clinical Trial Registration* NCT02319187. Registered on December 9, 2014

**Electronic supplementary material:**

The online version of this article (10.1186/s40880-019-0359-7) contains supplementary material, which is available to authorized users.

## Background

Esophageal cancer is the sixth most common type of malignancy in the world [1]. Esophageal squamous cell carcinoma (ESCC) is the predominant histological type of esophageal cancer in Eastern Europe and Asia. The use of platinum plus paclitaxel or fluorouracil-based chemotherapy are the preferred regimens for the first-line treatment of esophageal cancer, but despite such, the overall survival (OS) of these patients remains less than 1 year [2].

The clinical benefits of irinotecan [3], docetaxel [4], and ramucirumab [5] as second-line regimens for advanced esophageal adenocarcinoma have been validated in phase 3 trials, but the second-line treatment options for advanced ESCC patients remain investigational. The benefit of second-line targeted treatment in patients with advanced ESCC is still uncertain, and only a few treatment options are available [3]. The Cancer Oesophagus Gefitinib (COG) trial [4], which included 50 patients with ESCC in the gefitinib arm, is the largest randomized phase 3 trial performed for esophageal cancer patients whose disease progressed after chemotherapy. In that study, the gefitinib arm demonstrated a modest antitumor efficacy with an overall response rate (ORR) of 2.7% and a disease control rate (DCR) of 24%, and the observed median progression-free survival (PFS) and OS were 1.57 months and 3.73 months, respectively. Although no OS benefit from gefitinib over placebo was observed, a small benefit in PFS (1.57 vs. 1.17 months, *P* = 0.020) was achieved. By comparison, in another phase 2 trial, when pretreated ESCC patients having epidermal growth factor receptor (EGFR) overexpression or amplification were treated with icotinib, an EGFR-tyrosine kinase inhibitors (TKI), they demonstrated an ORR of 16.7% and a median PFS of 52 days [5]. It is noteworthy that despite the increased response rate in selected patients observed in the latter trial, the median PFS was still disappointing. Recently, the effect of immune checkpoint inhibitors on ESCC have also been evaluated in clinical trials, with ORR ranging between 17% and 33% and median PFS between 1.5 and 3.6 months [6–8]. However, only a small number of patients (*n* = 18–65) with ESCC were included in these phase 1/2 trials, and no standard treatments were established. As such, chemotherapy still remains the cornerstone in the treatment of advanced ESCC.

The combination of docetaxel with platinum has demonstrated a response rate of 25%–34.2% in metastatic ESCC patients whose disease progressed after first-line chemotherapy on platinum plus fluorouracil [9–11]. S-1, a fluoropyrimidine derivative, has been reported in a phase 2 trial as a second-line chemotherapy for patients with unresectable and recurrent ESCC, resulting in a median OS of 330 days [12]. Irinotecan, a topoisomerase I inhibitor, has demonstrated moderate activity as a single agent in metastatic gastro-esophageal cancer [13]. In our previous retrospective single institution study, we have observed a response rate of 29.6% in recurrent or metastatic ESCC patients treated with irinotecan plus a fluorouracil derivative as a second-line or third-line chemotherapy [14]. However, since the treatment options for recurrent or metastatic ESCC is still limited and the reported results have not shown significant improvement in patients’ survival, in this open-labeled phase 3 ESWN 01 trial, we intended to explore the benefit of adding irinotecan to S-1 as a second- or third-line chemotherapy for recurrent or metastatic ESCC.

## Patients and methods

### Patient selection and study design

The ESWN 01 was a prospective randomized, multicenter, open-label, phase 3 trial performed at 15 participating centers across China. The inclusion criteria were as follows: (i) patients between the age 18–70 years, (ii) had histological confirmation of metastatic or recurrent ESCC (staged using the seventh edition of the American Joint Committee on Cancer [AJCC] staging manual since enrolment ended in July 2016), (iii) had disease progression after platinum-based or taxane-based chemotherapy, (iv) had an Eastern Cooperative Oncology Group (ECOG) performance status of 0 to 2, (v) had measurable disease as per the Response Evaluation Criteria in Solid Tumors version 1.1 (RECIST version 1.1), and (vi) had adequate marrow and organ functions (assessed by the white blood cell, neutrophil, and platelet counts, measurement of hemoglobin concentration, and liver and kidney function tests). Patients were excluded if they had (i) previous treatments with irinotecan, fluorouracil, or any targeted or immunotherapy agents in a palliative setting, (ii) fluorouracil-based adjuvant chemotherapy within 6 months before the randomization, and (iii) cerebral or meningeal metastases. The data of patients’ prior lines of chemotherapy were collected.

The protocol was approved by the institutional review board and ethics committee at each institution. All recruited patients provided written informed consent before enrolment. This trial was performed in accordance with the principles of the Declaration of Helsinki and the Good Clinical Practice Guidelines of the International Conference on Harmonization and was registered at ClinicalTrials.gov, identifier: (identifier: NCT02319187).

### Randomization and masking

Patients were randomly allocated in a 1:1 ratio to either the irinotecan plus S-1 group or the S-1 monotherapy group, using a central computerized minimization procedure. The randomization was stratified by age (> 65 vs. ≤ 65 years), ECOG performance status (0–1 vs. 2), tumor differentiation (poorly vs. moderately/well differentiated), and the extent of disease (recurrent vs. metastatic). The patients, investigators, and other trial staff were all aware of the treatment allocations after randomization.

### Treatment procedures

In the irinotecan plus S-1 group, the patients received intravenous infusion of irinotecan (Jiangsu Hengrui Medicine Co. Ltd, Jiangsu, P.R. China) 160 mg/m^2^ on day 1, followed by oral S-1 (Jiangsu Hengrui Medicine Co. Ltd) 80–120 mg/day, on days 1–10, repeated every 14 days. In the S-1 monotherapy group, oral S-1 was prescribed at 80–120 mg/day on days 1–14, repeated every 21 days. The treatment doses and schedule in this study were based on the efficacy and safety data from our previous retrospective study on ESCC [14]. All patients received the allocated treatment until disease progression, intolerable adverse events, or withdrawal of consent.

Dose modifications due to treatment-related adverse events were allowed in this trial. If any patient developed grade 3 thrombocytopenia or other grade 4 hematologic adverse events related to the use of irinotecan, as assessed by the investigator, the dose of irinotecan was reduced by 20 mg/m^2^ each time to a minimum of 120 mg/m^2^ in the successive cycles. For patients developing grade 3 diarrhea related to irinotecan, the dose of irinotecan was reduced by 20 mg/m^2^ each time to a minimum of 100 mg/m^2^. For patients developing grade 3 nausea or diarrhea related to S-1, the dose of S-1 was reduced by 20 mg in the successive cycle. Granulocyte colony-stimulating factor (G-CSF) was administered to those who developed grade 3/4 neutropenia or leucopenia, whereas the prophylactic use of G-CSF was not allowed. Other anti-tumor treatments were not permitted during the study period. The post-study treatment (palliative radiation, systemic treatments) to be provided for those patients who experienced treatment failure was decided by the physician in charge at each respective institution and was not preplanned in the study design.

### Treatment follow-up and safety

Scheduled visits and computed tomography (CT) scans of the chest and abdomen were repeated every 6 weeks until the onset of progressive disease. The tumor response was assessed by independent central radiologic review based on the RECIST criteria, version 1.1.

Treatment safety was monitored throughout the treatment and for 30 days after the last prescribed study dose. Symptoms developed during the study treatment were recorded, and physical examinations were performed at each scheduled visit. A complete blood count (CBC) test was repeated every week, and a blood chemistry test was repeated every month starting from the first dose of the study drug to detect potential adverse events and guide dose modifications as per the study protocol. Other clinical tests including urine, fecal occult blood, coagulation, and electrocardiogram were repeated when necessary as decided by the investigators. All adverse events were graded according to the National Cancer Institute Common Terminology Criteria for Adverse Events (NCI-CTCAE; version 4.02). After completion of the treatments, patients were followed up every 8 weeks until death. The post-study follow-up data were also collected.

### Study outcomes

The primary endpoint was progression-free survival (PFS), defined as the time from randomization to radiographic progression, or death from any cause. Secondary endpoints included objective response rate (ORR), defined as the percentage of patients who had the best treatment response, either a radiological complete response or partial response; disease control rate (DCR), defined as the percentage of patients who had the best response of complete response, partial response, or stable disease. Other secondary endpoints included the duration of response, defined as the time from the first documentation of complete or partial response to radiological disease progression, and OS, defined as the time from randomization to death from any cause.

### Statistical analysis

Before the start of the study, we assumed that patients were to be recruited over an 18-month period and were to be followed-up for a minimum of 6 months. To achieve a 90% power to detect an improvement in PFS from 2.5 months in the S-1 monotherapy group to 4.0 months in the irinotecan plus S-1 group, by accounting for a 20% loss due to follow-up with a two-sided α of 0.05, 228 patients had to be randomly assigned. The primary endpoint was analyzed in the intention-to-treat population, defined as all enrolled patients who were randomly assigned to a group, regardless of whether they received the study treatment.

Chi square or Fisher’s exact tests were used to compare the patient characteristics, ORRs, and DCRs. Intention-to-treat analyses were carried out on eligible patients. PFS and OS were estimated by the Kaplan–Meier method and compared by the log-rank test. The hazard ratios (HRs) and 95% confidence intervals (CIs) were estimated with Cox proportional hazards model. Subgroup analyses of PFS were compared between the treatment groups using the Cox proportional hazard model. All statistical analyses were conducted using the SAS 9.4 software (SAS Institute Inc., Cary, NC, USA), and a *P* < 0.05 was considered statistically significant. Forest plot was created using Microsoft Excel 2010 (Microsoft Corporation, Redmond, WA, USA).

## Results

### Patient characteristics

**Fig. 1:**
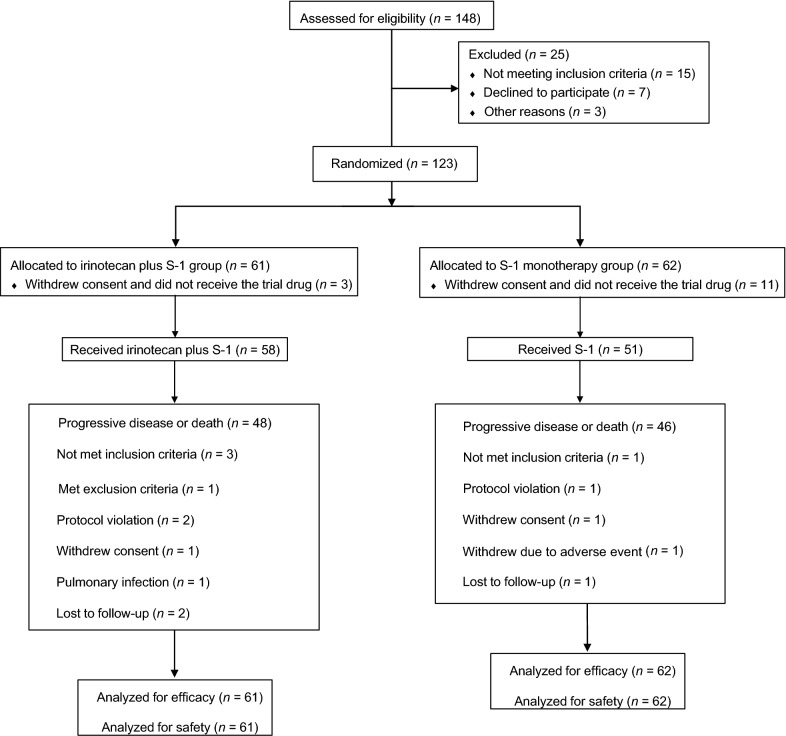
CONSORT diagram illustrating the design of the present study

Between December 23, 2014 and July 25, 2016, a total of 148 patients were screened for enrollment, of which 25 patients were excluded. The study design is illustrated in Fig. 1. Due to difficulty in recruiting patients, we had to stop recruitment in August 2016 but all patients were followed-up for at least 12 months to ensure data maturity. Of the 123 patients who were randomized and included in the intention-to-treat population, 61 patients were assigned to the irinotecan plus S-1 group and 62 patients to the S-1 monotherapy group. One hundred and nine patients received at least one dose of treatment. All enrolled patients were included in the efficacy and safety analysis. Two patients in the irinotecan plus S-1 group and one in the S-1 monotherapy group were lost to follow-up. The baseline characteristics of the 123 treated patients are listed in Table 1. The median age of the enrolled patients was 58.5 years (range 39.1–70.0 years). One hundred and twelve enrolled patients (91.1%, 112/123) had an ECOG performance status score of 0–1. All patients received platinum-based or taxane-based chemotherapy in the first-line setting. Forty-seven patients (38.2%, 47/123) had surgery and 64 (52.0%, 64/123) had received local radiation in previous treatments. Additionally, a large proportion of patients had at least one of the poor prognostic factors, including poorly differentiated tumors (46.3%, 57/123) and 3 or more metastatic sites (16.3%, 20/123). The baseline characteristics were generally well balanced between the two treatment groups.

**Table 1:** Baseline characteristics of the 123 treated patients with advanced esophageal squamous cell carcinoma (intention-to-treat population)

Characteristic	Treatment groups		*P* value
	Irinotecan plus S-1 [cases (%)]	S-1 monotherapy [cases (%)]	
Age			0.877
Median years (range)	60 (39.1–70.0)	57 (42.0–70.0)	
≤ 65 years	46 (75.4)	46 (74.2)	
> 65 years	15 (24.6)	16 (25.8)	
Sex			0.774
Male	56 (91.8)	56 (90.3)	
Female	5 (8.2)	6 (9.7)	
ECOG performance score			0.593
0	23 (37.7)	18 (29.0)	
1	33 (54.1)	38 (61.3)	
2	5 (8.2)	6 (9.7)	
Tumor grade			0.792^#^
Poorly differentiated	28 (45.9)	29 (46.8)	
Moderately differentiated	29 (47.5)	31 (50.0)	
Well differentiated	4 (6.6)	2 (3.2)	
Number of metastatic sites			0.409
1	23 (37.7)	19 (30.6)	
≥ 2	38 (62.3)	43 (69.4)	
Previous treatment^a^			
Surgery	28 (45.9)	19 (30.6)	0.082
Local radiation	32 (52.5)	32 (51.6)	0.925
Disease status			1.000^#^
Local recurrence	3 (4.9)	4 (6.5)	
Distant metastasis	58 (95.1)	58 (93.5)	
Prior lines of chemotherapy			0.668
First-line	51 (83.6)	50 (80.6)	
Second-line	10 (16.4)	12 (19.4)	

*ECOG* Eastern Cooperative Oncology Group

^#^Tested by Fisher’s exact test

^a^Some of the patients presented with metastatic disease at their initial diagnosis, therefore they did not receive either surgery or radiation

### Antitumor activity

At the end of follow-up (January 16, 2018), for a median follow-up duration of 29.2 months (95% CI 17.5–40.9 months), 96 (78.0%) patients had died. The median duration of treatment was 69 days (range 0–434 days) in the irinotecan plus S-1 group versus 36 days (range 0–662 days) in the S-1 monotherapy group.

**Fig. 2:**
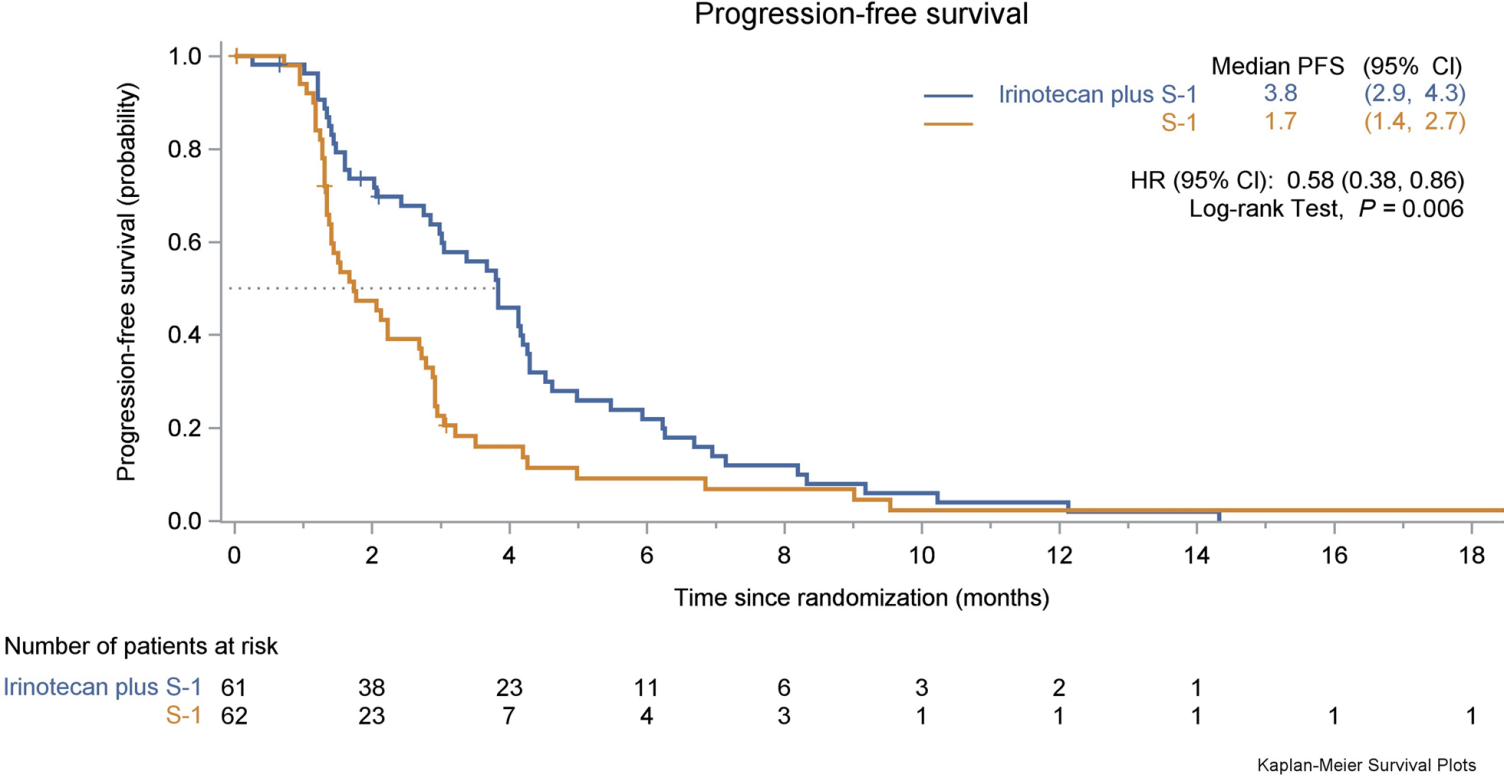
Kaplan–Meier estimate of the progression-free survival in the two treatment groups. Since there was one patient in the S-1 monotherapy group who had a long duration of response (21.7 months), the PFS curve was thus longer for the S-1 group. However, the median PFS was longer in the irinotecan plus S-1 group than in the S-1 monotherapy group. *PFS* progression free survival, *CI* confidence interval, *HR* hazard ratio

**Fig. 3:**
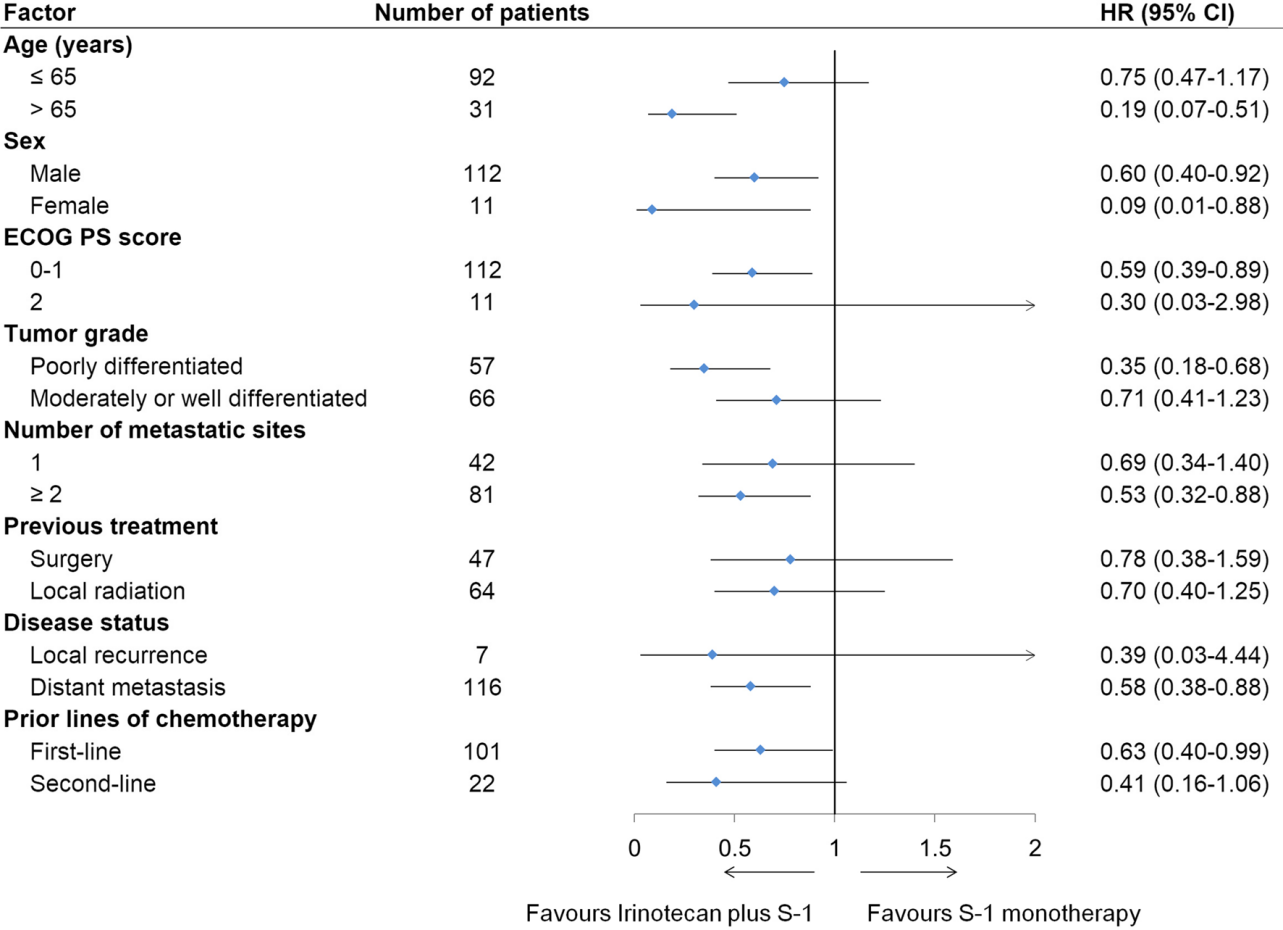
Forest plot illustrating the progression-free survival in prespecified subgroups. The arrows, above the scale, pointing to the right indicates that the upper limit of the 95% CI of HR exceeds 2. *HR* hazard ratio, *CI* confidence interval, *ECOG PS* Eastern Cooperative Oncology Group performance score

Based on the 99 observed events of disease progression or death at the time of this report, PFS was significantly longer in the irinotecan plus S-1 group than in the S-1 monotherapy group (HR = 0.58, 95% CI 0.38–0.86, *P *= 0.006; Fig. 2). The median PFS was 3.8 months (95% CI 2.9–4.3 months) in the irinotecan plus S-1 group compared with 1.7 months (95% CI 1.4–2.7 months) in the S-1 monotherapy group. The median duration of response was 4.0 months (95% CI 2.2–5.9 months) in the irinotecan plus S-1 group and 2.9 months (95% CI 0.3–5.3 months) in the S-1 monotherapy group. Further details regarding the duration of disease control of both treatment groups are provided in Additional file 1: Figure S1. The 6-month PFS rate was 21.9% in the irinotecan plus S-1 group and 9.1% in the S-1 monotherapy group, respectively. The benefit of adding irinotecan to S-1 monotherapy in PFS was observed in most of the examined subgroups (Fig. 3).

**Fig. 4:**
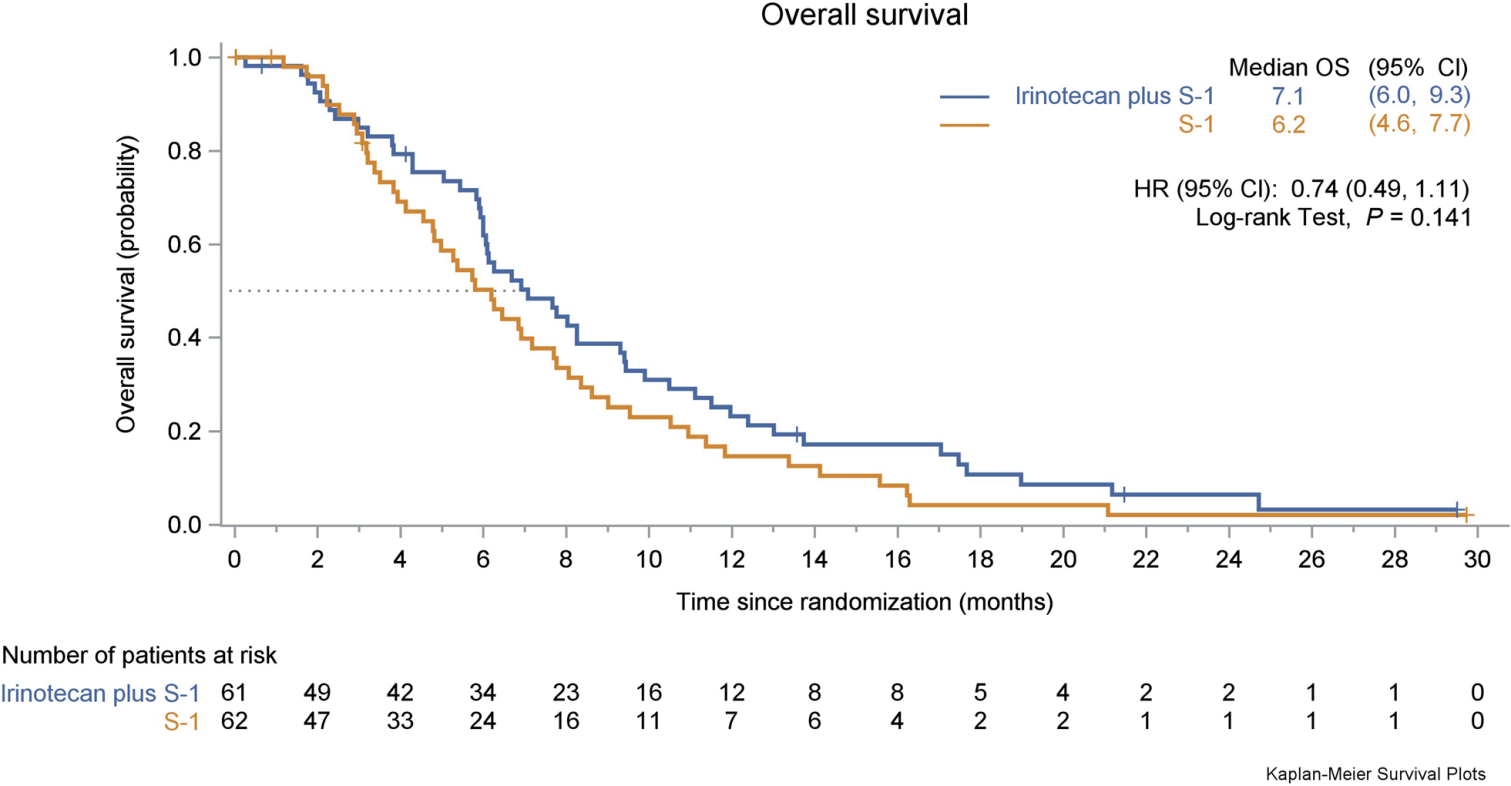
Kaplan–Meier estimate of the overall survival for the two treatment groups. *OS* overall survival, *CI* confidence interval, *HR* hazard ratio

The median OS was 7.1 months (95% CI 6.0–9.3 months) in the irinotecan plus S-1 group and 6.2 months (95% CI 4.6–7.7 months) in the S-1 monotherapy group (HR = 0.74, 95% CI 0.49–1.11, *P *= 0.141; Fig. 4). The 6-month OS rate was 61.9% in the irinotecan plus S-1 group and 50.2% in the S-1 monotherapy group, respectively.

All patients who were randomized were included in the response analysis (Table 2). The ORR was 24.6% (15/61) in the irinotecan plus S-1 group, compared with 9.7% (6/62) in the S-1 monotherapy group (*P* = 0.002). Complete response was observed in one of the 61 patients in the irinotecan plus S-1 group and none of the 62 patients in the S-1 monotherapy group. The DCR was significantly higher in the irinotecan plus S-1 group than the S-1 monotherapy group (57.3% vs. 35.4%, *P* < 0.001).

**Table 2:** Tumor response of the investigated patients with advanced esophageal squamous cell carcinoma as determined based on CT scans

Response	Treatment groups	
	Irinotecan plus S-1 [cases (%)]	S-1 monotherapy [cases (%)]
Total	61	62
CR	1 (1.6)	0 (0.0)
PR	14 (22.9)	6 (9.7)
SD	20 (32.8)	16 (25.8)
PD	14 (23.0)	26 (41.9)
Not assessable	12 (19.7)	14 (22.6)

*CT* computed tomography, *CR* complete response, *PR* partial response, *SD* stable disease, *PD* progressive disease

A higher proportion of patients in the S-1 monotherapy group received subsequent chemotherapy after the present trial (22/62 [35.48%] in the S-1 monotherapy group vs. 8/61 [13.1%] in the irinotecan plus S-1 group), and a total of 14 patients in the S-1 monotherapy group received irinotecan-based chemotherapy after disease progression. The proportion of patients receiving anti-PD-1 antibodies in later lines of treatment were almost identical (4/62 [6.4%] in the S-1 monotherapy group vs. 4/61 [6.6%] in the irinotecan plus S-1 group). There were 2 patients, one from the irinotecan plus S-1 group and one from the S-1 monotherapy group whose tumor demonstrated PR on the PD-1 treatment. Further, there was one patient from the irinotecan plus S-1 group who had CR.

### Treatment safety

The intention-to-treat population was assessed for adverse events. The most common treatment-related adverse events were leukopenia (34/61, 55.7%), anemia (33/61, 54.1%), and neutropenia (29/61, 47.5%) in the irinotecan plus S-1 group and were fatigue (22/62, 35.5%), anemia (20/62, 32.3%), leukopenia (15/62, 24.2%), and anorexia (15/62, 24.2%) in the S-1 monotherapy group. The rates of grade 3 or 4 adverse events are illustrated in Table 3. No severe adverse events were observed, and no patients were hospitalized for any of the documented adverse events in the study. The addition of irinotecan was associated with increased rates of grade 3–4 leukopenia and neutropenia but there was no significant difference in grade 3–4 diarrhea between the two treatment groups and no patient died from any of the observed adverse events.

**Table 3:** Summary of treatment-related grade 3 or 4 adverse events in the intention-to-treat population

Adverse events (grade 3 or 4)	Total patients [cases (%)] (*N* = 123)	Treatment groups		*P* value^#^
		Irinotecan plus S-1 group [cases (%)] (*n* = 61)	S-1 monotherapy group [cases (%)] (*n* = 62)	
Anemia	3 (2.4)	2 (3.3)	1 (1.6)	0.619
Leukopenia	10 (8.1)	10 (16.4)	0 (0)	0.001
Neutropenia	10 (8.1)	9 (14.8)	1 (1.6)	0.008
Thrombocytopenia	2 (1.6)	2 (3.3)	0 (0)	0.496
Diarrhea	3 (2.4)	2 (3.3)	1 (1.6)	0.619
Nausea	3 (2.4)	3 (4.9)	0 (0)	0.119
Vomiting	2 (1.6)	1 (1.6)	1 (1.6)	0.748
Fatigue	3 (2.4)	2 (3.3)	1 (1.6)	0.619
Anorexia	1 (0.8)	0 (0)	1 (1.6)	1.000
Elevated bilirubin	1 (0.8)	1 (1.6)	0 (0)	1.000

^#^Tested by Fisher’s exact test

## Discussion

To the best of our knowledge, this is the first prospective, randomized, controlled study comparing doublet with single-agent chemotherapy in patients with advanced ESCC progressing after systemic cytotoxic therapies. Patients treated with irinotecan plus S-1 demonstrated a significantly longer PFS and higher response rate than did those treated with S-1 monotherapy. The treatments were well tolerated in both groups. The toxicity profile observed in this study was generally consistent with that observed in other tumor types [15, 16].

A total of 15 centers across China registered to participate in this study, but only 148 patients could be screened. Patient accrual was difficult partly due to the open-label design of the trial. The most frequent reason for refusal to enroll, as reported from the participating centers, was the unwillingness of the patients to receive single-agent treatment. There were also 14 cases (3 in the irinotecan plus S-1 group and 11 in the S-1 monotherapy group) of early withdrawal of consent after randomization.

Despite the promising breakthrough in immunotherapy, cytotoxic chemotherapy still remains the mainstream in the management of recurrent or metastatic ESCC patients. However, no randomized phase 2 or 3 trials have been reported for patients with recurrent or metastatic ESCC who had resistance to platinum- or taxane-based chemotherapy. Currently, S-1 or fluorouracil, platinum, taxanes, and irinotecan are the accepted drugs for these patients [17]. The combination of irinotecan with fluorouracil or capecitabine has demonstrated activity in patients with metastatic esophageal cancer progressing after platinum-based first-line chemotherapy [15, 16]. However, these phase 2 trials included both ESCC and esophageal adenocarcinoma patients, and the number of patients enrolled was limited. In this ESWN 01 trial, which enrolled only ESCC patients, the results showed that treatment with irinotecan plus S-1 significantly prolonged PFS as compared with S-1 alone, and the observed treatment outcomes can be regarded clinically meaningful. The ORR and DCR were also higher in the irinotecan plus S-1 group than in the S-1 monotherapy group (ORR: 24.6% vs. 9.7% [*P* = 0.002]; DCR: 57.3% vs. 35.4% [*P* < 0.001]). Both the ORR and median PFS of the irinotecan plus S-1 regimen in the present study were numerically slightly inferior to the findings observed in our previous retrospective study [14], nevertheless, the efficacy of the doublet regimen from these two studies were comparable even though the study design and patient population were different. The addition of irinotecan did not significantly prolong the median OS in the present study (irinotecan plus S-1 group [7.1 months] vs. S-1 monotherapy group [6.2 months], *P *= 0.141), despite an observed superior 6-month OS rate in the irinotecan plus S-1 group (61.9% vs. 50.2%, respectively). This could have been partially attributed due to a substantial number of patients in the S-1 monotherapy group who received irinotecan or irinotecan-based chemotherapy in subsequent lines of treatment after failure with S-1 monotherapy. Another reason could have been the decreased willingness of the patients in the irinotecan plus S-1 group to undergo further chemotherapy when their diseases progressed beyond the study treatment for the fear of severe adverse events. In this prospective randomized setting, our results substantiate the role of the irinotecan plus S-1 regimen as a possible important alternative regimen for patients with recurrent or metastatic ESCC. Our future direction could be exploring the combination of irinotecan plus S-1 chemotherapy with immune checkpoint inhibitors or other targeted treatments.

The regimens used in both groups of the present study appeared to be well tolerated. Irinotecan and S-1 have distinct toxicity profiles. In this study, more patients developed grade 3–4 neutropenia, leucopenia, and nausea in the irinotecan plus S-1 group. Despite a higher rate of adverse events in the irinotecan plus S-1 group, only a few patients discontinued the treatment due to the adverse events. Several phase 2 trials evaluated the efficacy of irinotecan-based chemotherapy in esophageal or esophago-gastric cancer patients in which the dosage schedules of irinotecan were 180 mg/m^2^ repeated every 2 weeks and 250 mg/m^2^ repeated every 3 weeks respectively, and the reported rates of grade 3–4 neutropenia ranged between 26.4% and 31% [15, 16], whereas the rate of common adverse events in the irinotecan plus S-1 group of the present study was much lower, at 14.8%. Similarly, the rate of grade 3–4 diarrhea was lower in the irinotecan plus S-1 group (3.3%) than those reported in the two above-mentioned trials (7.9%–15.0%) [15, 16]. A possible explanation for this difference in observed adverse events could have been due to the lower dose of irinotecan (160 mg/m^2^) used in our study.

The present study had several limitations. First, the randomized controlled trial was prematurely stopped due to the stagnation in subject recruitment. As a result, the number of randomized patients was smaller than proposed in the protocol. Second, the open-labeled design of this trial had led to a certain number of patients who were randomized (*n* = 123) but did not receive the study treatment (*n* = 11) in the S-1 monotherapy group. This could have partly affected the evaluation of the efficacy of S-1.

## Conclusions

The addition of irinotecan to S-1 monotherapy in treating patients with recurrent or metastatic ESCC in the second- or third-line setting may prolong PFS and increase ORR, and can therefore be considered an alternative option as to S-1 monotherapy in this patient population.

## Additional file

**Additional file 1: Figure S1.** Illustration of the duration of disease control in (A) irinotecan plus S-1 group (*n* = 35) and (B) S-1 monotherapy group (*n* = 22). The number of horizontal blue bars represents the number of patients in each group and the length of each bar represents the follow-up period. The bars without symbols of clinical response represent the patients with stable disease throughout the study period. Abbreviations: CR, complete response; PR, partial response; PD, progressive disease

## Abbreviations

ESCC: esophageal squamous cell carcinoma
PFS: progression-free survival
CI: confidence interval
OS: overall survival
ECOG: Eastern Cooperative Oncology Group
RECIST: Response Evaluation Criteria in Solid Tumors
NCI-CTCAE: National Cancer Institute Common Terminology Criteria for Adverse Events
ORR: objective response rate
DCR: disease control rate
HRs: hazard ratios
CR: complete response
PR: partial response
